# The "Offa" Ventilator

**Published:** 1902-05-17

**Authors:** 


### THE "OFFA" VENTILATOR.

It is frequently difficult to ventilate a small ward, or room in a private house, without causing a draught to the patient, and this, unless the open-air treatment is being carried to excess, is obviously undesirable. Moreover, in crowded cities it is not draughts only that enter through open windows; dust, smuts from the neighbouring chimneys, and all kinds of poisonous particles may float in as well, and any system of ventilation by which this can be obviated merits attention.

It cannot be claimed that the "Offa" ventilation system is perfect as far as the "straining" of the air is concerned, but the idea is a good one, and one that may possibly be improved upon. The principle is simple, and, as we have seen it demonstrated at the offices of the "Offa" Company, effective. It is arrived at in the following way: You first knock out one or more bricks in the wall of your house and substitute, on the outer side, perforated bricks; next you fix a frame covered with a strainer made of an open-work duster, into the opening on the inner side; and, lastly, you punch two holes, one on each side of the opening, in which the two screws of a metal shield may run; the shield is provided with a knob, and pulls out or shuts up tight. In other words, take any ordinary pantry window covered with wire gauze; the draught blows too directly into the room, say on the head of the person washing up. To obviate this you fix a square of metal in front, slightly larger than the size of the window, and the draught is diffused—above, below, and on either side. Ornament the metal covering in bronze repoussé or any other style, and it will be fit for the drawing-room; use a plain black enamelled or polished shield and you have a useful ventilating apparatus for a public institution, workshop, or any other building where it is desirable to admit a quantity of fresh air but to avoid draughts. One of the advantages of this form of ventilator is that it is silent; there is no loose grating to cause an annoying rattle when the wind is blowing. It is also clean, since the "air-strainer" is made, as described above, of an open-work duster stretched on a frame, which can be taken out, washed, and even renewed quite easily and cheaply. In a clean country place it might not be necessary to use this at all. Another point to observe is that the ventilator need not show; it may be behind a large picture, and no one be the wiser unless sitting directly underneath it. Moreover, the shield can be closed so as to admit no air, if required.

The "Offa" is made in five sizes, and the external openings required correspond with brick dimensions, that is, from 9 inches by 6 to 18 by 18.

These ventilators were introduced in 1895 to one of Messrs. Huntley and Palmer's bakeries, Reading. The address of the "Offa" Company is 64 Basinghall Street, London, E.C.

